# Anti-arrhythmic Cardiac Phenotype Elicited by Chronic Intermittent Hypoxia Is Associated With Alterations in Connexin-43 Expression, Phosphorylation, and Distribution

**DOI:** 10.3389/fendo.2018.00789

**Published:** 2019-01-25

**Authors:** Jana Kohutova, Barbara Elsnicova, Kristyna Holzerova, Jan Neckar, Ondrej Sebesta, Jana Jezkova, Marek Vecka, Pavel Vebr, Daniela Hornikova, Barbara Szeiffova Bacova, Tamara Egan Benova, Marketa Hlavackova, Narcis Tribulova, Frantisek Kolar, Olga Novakova, Jitka M. Zurmanova

**Affiliations:** ^1^Department of Physiology, Faculty of Science, Charles University, Prague, Czechia; ^2^Developmental Cardiology, Institute of Physiology of the Czech Academy of Sciences, Prague, Czechia; ^3^Laboratory of Confocal and Fluorescence Microscopy, Faculty of Science, Charles University, Prague, Czechia; ^4^4th Department of Internal Medicine, 1st Faculty of Medicine, Charles University, Prague, Czechia; ^5^Center of Experimental Medicine of the Slovak Academy of Sciences, Institute for Heart Research, Bratislava, Slovakia

**Keywords:** heart, chronic hypoxia, brief ischemia, arrhythmia, connexin-43, n-3 PUFA

## Abstract

Remodeling of the cellular distribution of gap junctions formed mainly by connexin-43 (Cx43) can be related to the increased incidence of cardiac arrhythmias. It has been shown that adaptation to chronic intermittent hypobaric hypoxia (IHH) attenuates the incidence and severity of ischemic and reperfusion ventricular arrhythmias and increases the proportion of anti-arrhythmic n-3 polyunsaturated fatty acids (n−3 PUFA) in heart phospholipids. Wistar rats were exposed to simulated IHH (7,000 m, 8-h/day, 35 exposures) and compared with normoxic controls (N). Cx43 expression, phosphorylation, localization and n−3 PUFA proportion were analyzed in left ventricular myocardium. Compared to N, IHH led to higher expression of total Cx43, its variant phosphorylated at Ser368 [p-Cx43(Ser368)], which maintains “end to end” communication, as well as p-Cx43(Ser364/365), which facilitates conductivity. By contrast, expression of non-phosphorylated Cx43 and p-Cx43(Ser278/289), attenuating intercellular communication, was lower in IHH than in N. IHH also resulted in increased expression of protein kinase A and protein kinase G while casein kinase 1 did not change compared to N. In IHH group, which exhibited reduced incidence of ischemic ventricular arrhythmias, Cx43 and p-Cx43(Ser368) were more abundant at “end to end” gap junctions than in N group and this difference was preserved after acute regional ischemia (10 min). We further confirmed higher n-3 PUFA proportion in heart phospholipids after adaptation to IHH, which was even further increased by ischemia. Our results suggest that adaptation to IHH alters expression, phosphorylation and distribution of Cx43 as well as cardioprotective n-3PUFA proportion suggesting that the anti-arrhythmic phenotype elicited by IHH can be at least partly related to the stabilization of the “*end to end”* conductivity between cardiomyocytes during brief ischemia.

## Introduction

Proper intercellular communication is essential for normal electrical activation of the myocardium and synchronized contraction of the heart for [review see (1)]. Various pathological states may lead to rhythm disturbances due to altered intercellular communication resulting in changes of conduction properties, expression and distribution of connexin-43 (Cx43), as a main component of gap junction (GJ) channels within ventricular myocardium (2, 3). Reduction of GJ number and conductance increases susceptibility to spontaneous and inducible ventricular arrhythmias both under control conditions and during ischemia and reperfusion (I/R) (4, 5). Beside the number and properties of GJs, the location of Cx43 is also an important aspect contributing to arrhythmogenesis [reviewed by (3, 6, 7)].

GJ permeability and conductivity are controlled by complex and multifactorial processes. These events are generally influenced by changes of intracellular environment under physiological and pathological conditions, e.g., pH and Ca^2+^ concentration (8–10). Specifically, the GJ conductivity is strongly influenced by Cx43 expression levels, its posttranslational modifications, protein-protein interactions, e.g., dimerization and interaction with Zona Occludens (ZO-1), Cx43 assembly and last but not least, the size of GJ plaques and their localization within cardiomyocytes [for review see (11)].

Under physiological conditions, GJs are predominantly located at the intercalated disks providing “*end to end”* conduction between neighboring cardiomyocytes. Small amounts of Cx43 are also found in the lateral plasma membrane away from the intercalated disks, allowing lateral conduction between cardiomyocytes (i.e., “*side to side”* conduction). Decreased expression of Cx43 as well as increased “*side to side”* conduction can cause deceleration and abnormal conduction leading to the generation of arrhythmias (12). On the other hand, ischemic preconditioning delayed electrical uncoupling and Cx43 de-phosphorylation (13).

Various modes of chronic hypoxia are well known to induce adaptive responses improving cardiac tolerance to major manifestations of acute I/R injury. It has been shown repeatedly that hearts adapted to chronic intermittent hypoxia (IHH) exhibit smaller infarct size, improved recovery of contractile function and, in particular, lower propensity to ventricular arrhythmias occurring during I/R insult (14–18). Importantly, we demonstrated previously that adaptation to IHH increases the abundance of antiarrhythmic n-3 polyunsaturated fatty acids (n-3 PUFA) in heart phospholipids (19). Although multiple factors have been shown to play a role in this form of cardioprotection (20, 21), the detailed mechanism is still unclear. To our knowledge, the potential involvement of Cx43 in the anti-arrhythmic effect of IHH has not been investigated.

Therefore, the goal of the present study was to assess the expression, phosphorylation and distribution of Cx43 as well as the expression of Cx43 upstream kinases in the myocardium of rats adapted to IHH. Moreover, the distribution of Cx43/p-Cx43(Ser368) between “*end to end”* and “*side to side”* GJs as well the proportion of antiarrhythmic n-3 PUFA in heart phospholipids following brief ischemia were analyzed.

## Materials and Methods

### Animal Model

Adult (8-week-old) male Wistar rats (250–280 g body weight) were exposed for 5 weeks to simulated IHH for 8-h per day, 5 days per week. Barometric pressure (*P*_B_) in the chamber was lowered stepwise, so that the level equivalent to an altitude of 7,000 m (*P*_B_ = 308 mm Hg, 41 kPa; *P*O_2_ = 65 mm Hg, 8.6 kPa) was reached after 13 exposures. A control group was kept in the chamber under normoxic conditions equivalent to an altitude of 200 m (*P*_B_ = 742 mm Hg, 99 kPa; *P*O_2_ = 155 mm Hg, 20.7 kPa) for the same period of time. Rats were fed by standard laboratory diet and kept at the 12/12-h light/dark cycle. Body weight of rats at the end of experiment increased to 407 ± 54 g and 505 ± 47 g in IHH and normoxic groups, respectively. The maintenance and handling of experimental animals was in accordance with the Guide for the Care and Use of Laboratory Animals published by the US National Institutes of Health (NIH Publication No. 85–23, revised 1996). The experimental protocol was approved by the Animal Care and Use Committee of the Institute of Physiology of the Czech Academy of Sciences.

### Regional Ischemia

For immunolocalization studies and lipid composition analyses, subgroup of hearts from each group was subjected to acute ischemia as described previously (22). Briefly, animals were anesthetized (sodium pentobarbital, 60 mg/kg i.p.) and ventilated with room air at 68–70 strokes/min (tidal volume of 1.2 ml/100 g body weight). A single-lead electrocardiogram was continuously recorded and subsequently analyzed by a custom-designed software. The rectal temperature was maintained between 36.5 and 37.5°C by a heated table throughout the experiment. Regional ischemia, the occlusion of the left coronary artery about 1–2 mm distal to its origin, was induced in open-chest rats after 15 min of stabilization by tightening the suture threaded through a polyethylene tube for 10 min. The aim was to obtain ischemic myocardium with fully reversible injury. Hearts of sham-operated rats not subjected to ischemia (the suture beneath the artery was not ligated) served as controls. Rats were killed by cervical dislocation and their hearts were rapidly excised, washed in ice-cold saline and processed for further analyses.

### Analysis of Arrhythmias

The incidence of ventricular arrhythmias during the 10-min ischemic episode was assessed as previously described (16). Premature ventricular complexes (PVCs) occurring as singles, salvos or tachycardia (a run of 4 or more consecutive PVCs) were counted separately. The incidence and duration of life-threatening ventricular tachyarrhythmias, i.e., tachycardia and fibrillation, were also evaluated.

### Immunofluorescence Study

#### Immunofluorescence Staining

Six hearts from each experimental group were used for the immunofluorescence localization studies following a previously described protocol (23). Briefly, hearts were fixed by perfusion with 4% paraformaldehyde for 2 min. Thereafter, hearts were immersed in buffered 4% formaldehyde for 2 h and subsequently incubated in 20% sucrose solution in PBS overnight. Separated left ventricles (LV) were cut transversally at one-third from apex and snap-frozen in liquid nitrogen. Longitudinal LV cryosections (6 μm, prepared on cryostat Leica CM3050, Leica-microsystems) were permeabilised in ice-cold methanol and then rinsed in 1% SDS as an antigen retrieval step. Non-specific binding sites were blocked for 1 h at room temperature using 10% donkey serum in PBS containing 0.3% Triton X-100, 1% BSA, 0.3 M glycine and unconjugated donkey anti-rabbit IgG (Sigma-Aldrich). Sections were incubated with rabbit monoclonal antibody against Cx43 (Sigma-Aldrich) or with rabbit polyclonal antibody against p-Cx43(Ser368) (Santa Cruz). Primary antibodies were detected with donkey anti-rabbit IgG conjugated with Alexa Fluor 488 (Thermo Fisher Scientific). Sections were incubated with Alexa Fluor 647 conjugated wheat germ agglutinin (WGA, Thermo Fisher Scientific) to stain plasma membranes and the tubular system. Sections were then mounted in ProLong Gold Antifade Reagent containing the nuclei marker 4′, 6-diamidino-2-phenylindole (DAPI) (Invitrogen) and stored at 4°C.

#### Image Acquisition

Samples were examined using an inverted fluorescence microscope (Olympus IX81, Olympus, Tokyo, Japan) equipped with a MT20 mercury arc illumination unit, fully motorized stage (Märzhäuser Wetzlar, Corvus) and CCD camera (Hamamatsu—Orca C4742-80-12AG). Five ROIs from each experimental sample were acquired with 20x NA1.2 Plan-Apochromat lens with zero gain and 1 × 1 binning in 1344 × 1024 format, 16-bit. Filter combination for individual channels were set as follows: DAPI—triple-band set 69002-ET-DAPI/FITC/TexasRed® (Chroma Technology Corp.) ex. 350 nm (bandwidth 50 nm) em. 457 nm (bandwidth 22 nm), connexin 43 (Alexa Fluor 488)—fl. cube U-MWIBA3 (Olympus), ex. 477.5 nm (bandwidth 17.5 nm) em. 530 nm (bandwidth 20 nm), WGA (TMR)—fl. Cube U-MWIGA3 (Olympus), ex. 540 nm (band-width 10 nm), em. 600 nm (bandwidth 25 nm).

#### Image Analysis

Images were processed using FIJI open source software (24). Automatic process of quantification was performed by a sequence of four steps: (i) *Image pre-processing*: Raw images were calibrated according to objective characteristics and acquisition mode (see above), resulting in pixel size of 0.3225 × 0.3225 μm. Uneven illumination was corrected using *Background subtraction* algorithm of FIJI ImageJ (created by Michael Castle and Janice Keller, https://imagej.net/Rolling_Ball_Background_Subtraction) with rolling ball radius set to 50 pixels. (ii) *Threshold setting*: For each image, threshold was manually set to select all Cx43 particles. The area of individual Cx43 particles was calculated and each particle was saved as ROI for further selection. (iii) *Cx43 particles determination:* WGA staining was used as marker of transversal/longitudinal orientation of the myocyte. A total of particles connecting myocytes in longitudinal course were distinguished as “*end to end”* type and junctions in transversal direction were defined as “*side to side”*. (iv) “*End to end” region:* The percentage of “*end to end*” junction area to the total junction area was calculated. A custom script was written to allow semi-automation of the process.

### Lipid Analysis

LV myocardium of normoxic and IHH rats subjected to brief ischemia and controls were frozen in liquid nitrogen and pulverized. Phospholipids were extracted from tissue samples according to the modified method of Folch et al. (25). Total phospholipids were separated by one-dimensional thin-layer chromatography using the solvent mixture hexane-ether-acetic acid (80:20:3). For FA analyses, phospholipid spots were visualized under UV light after staining with 0.005% 2′, 7′-dichlorofluorescein in methanol, scraped out and stored under nitrogen atmosphere at −20°C until the next day when methyl esters were prepared. For FA methyl ester preparation, sodium methanolate was added to tubes with silica gel and tubes were incubated for 60 min at room temperature in the dark. Methyl esters were extracted with hexane. The extract was evaporated under a stream of nitrogen and stored at −20°C. Methyl esters were separated using the gas chromatograph CP 438 A (Chrompack, Middelburg, The Netherlands) with the middle polar column CP WAX 52 CB (25 m × 0.25 mm i.d.). The oven temperature was programmed from 145 to 230°C at the rate of 2°C/min. Hydrogen was used as carrier gas. FA were identified using a mixture of FA methyl esters (26).

### SDS-PAGE and WB Analysis

The tissue was handled as described previously (23). The LVs were isolated, weighed, and immediately frozen in liquid nitrogen. Frozen tissue was pulverized in liquid nitrogen and subsequently homogenized in homogenization buffer [12.5 mM TRIS, 2.5 mM EGTA, 1 mM EDTA, 250 mM sucrose, 5 mM DL-dithiothreitol (DTT), protease and phosphatase inhibitor cocktail mix (Roche/Sigma-Aldrich), pH 7.4]. Homogenates were diluted by urea buffer in the ratio 1:1 for proper solubilization (5 M urea, 2 M thiourea, 0.4 M DTT, 10 mM sodium pyrophosphate tetrabasic decahydrate) and subsequently protein concentrations were measured using the Bradford dye binding assay (Bio-Rad). Homogenates were aliquoted and stored at −80°C for further analyses. Five homogenate samples from each group were separated by sodium dodecyl sulfate polyacrylamide gel electrophoresis on 10% bis-acrylamide gels at a constant voltage of 200 V using Mini-Protean TetraCell (Bio-Rad). The gel-resolved proteins were electro-transferred onto nitrocellulose membrane (0.2 μm pore size, Bio-Rad) at a constant voltage of 100 V for 1 h using a Mini Trans-Blot Module (Bio-Rad). Subsequently, membranes were blocked with 5% dry low fat milk in Tris-buffered saline with Tween 20 (TTBS) for 60 min at room temperature, washed in TTBS and individually incubated overnight at 4°C with the following primary antibodies: anti-GAPDH (Santa Cruz Biotechnology), anti-total-Cx43 (t-Cx43) (Sigma-Aldrich), anti-non-phospho-Cx43 (np-Cx43) (ThermoFisher), anti-phospho-Cx43 at Ser368 [p-Cx43(Ser368)] (Santa Cruz), anti-phospho-Cx43 at Ser279/282 [p-Cx43(Ser279/282)] (Santa Cruz), anti-phospho-Cx43 at Tyr265 p-Cx43(Tyr265) (Santa Cruz), anti-protein kinase A (PKA, Santa Cruz), anti-protein kinase G (PKG, Cell Signaling), anti-casein kinase 1 (CK1, Cell Signaling). Subsequently, membranes were washed in TTBS and incubated with the appropriate secondary antibodies: either anti-rabbit (GE Healthcare Amersham) or anti-mouse immunoglobulins (Thermo Fisher Scientific) conjugated with HRP, visualized by chemiluminescence (DuraSignal) using LAS-4000 imaging system (Genetica, Fujifilm) and quantified using Quantity One Software (Bio-Rad). The same amount of protein was loaded on the gels in the range of 10 to 30 μg per lane. All samples from each group were always run on the same gel and quantified on the same membrane. Glyceraldehyde 3-phosphate dehydrogenase and actin expression was used as loading control. Each sample was analyzed in duplicate in at least four gels. The results were normalized to total protein amount.

### Mass Spectrometry for Myocardial Cx43 Phosphorylation

Five hearts from normoxic and IHH groups were powdered and solubilized in 4% SDS, 10 mM TCEP, 40 mM chloroacetamide and 100 mM Tris, pH 7.5. Samples were kept 5 min at 95°C and sonicated 4-times for 20 s each. Samples were precipitated by addition of 4 volumes of cold acetone overnight (−20°C). The pellet was resuspended in 10% TFE (trifluorethanol) and 100 mM ammonium bicarbonate. Samples (500 μl) containing 2 mg of proteins were cleaved with trypsin overnight (27). Phosphopeptides were enriched according to Humphrey et al. (28). LC-MS was done on OrbitrapFusion coupled with NanoLC Dionex Ultimate 3000RS. A 50 cm Thermoeasy nano column connected in combination with upstream placed trap column 300 um ID, 5 mm length was used for separation. Two hours separation gradient was used. Data were searched with MaxQuant (29) software against latest Uniprot Rattus Norvegicus database with phospho STY like variable modification. Data from MaxQuant were further processed with Perseus Software (30). Quantitative values for identified phosphopeptides were subjected to further statistical analysis.

### Statistical Analysis

The statistical differences between the groups were determined by Two Way ANOVA with Bonfferoni post-test for immunofluorescence and fatty acid analyses. The incidence of tachyarrhythmias was examined by Fisher's exact test. The duration of tachyarrhythmias and the number of PVCs were compared by the Mann-Whitney U test. Unpaired *t*-test was used for all other experiments. Values of *p* < 0.05 were considered statistically significant. Data were expressed as a mean ± SEM.

## Results

### Myocardial Expression of Total Cx43 and Its Phosphorylated Status

**Figure 1:**
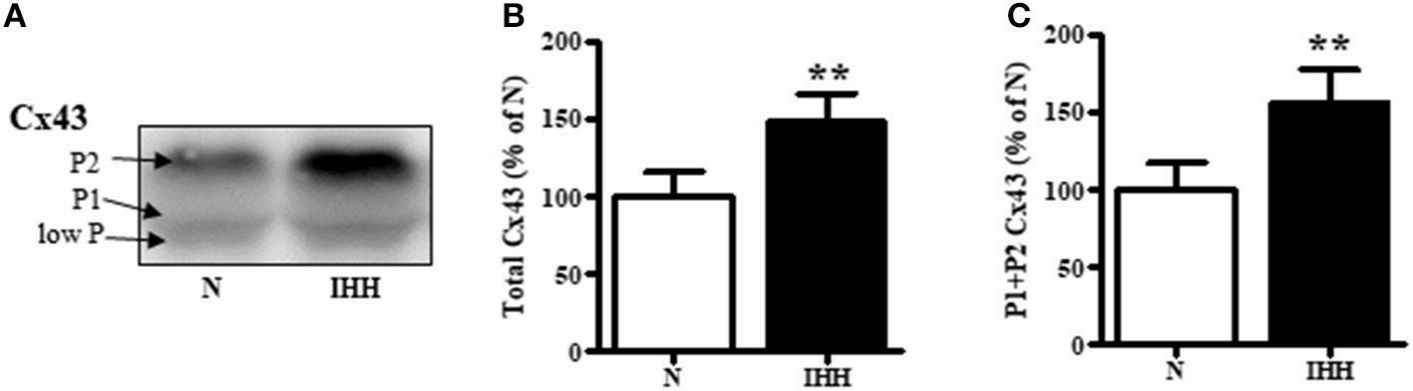
Effect of chronic intermittent hypobaric hypoxia (IHH, black columns) on expression of total Cx43 **(B)** and its high-phosphorylated forms (P1+P2 Cx43) **(C)** compared with normoxic group (N, open columns). The representative image of western blotting is shown **(A)**. Values are mean ± SEM, (*n* = 5 in each group), ^**^p < 0.01.

**Figure 2:**
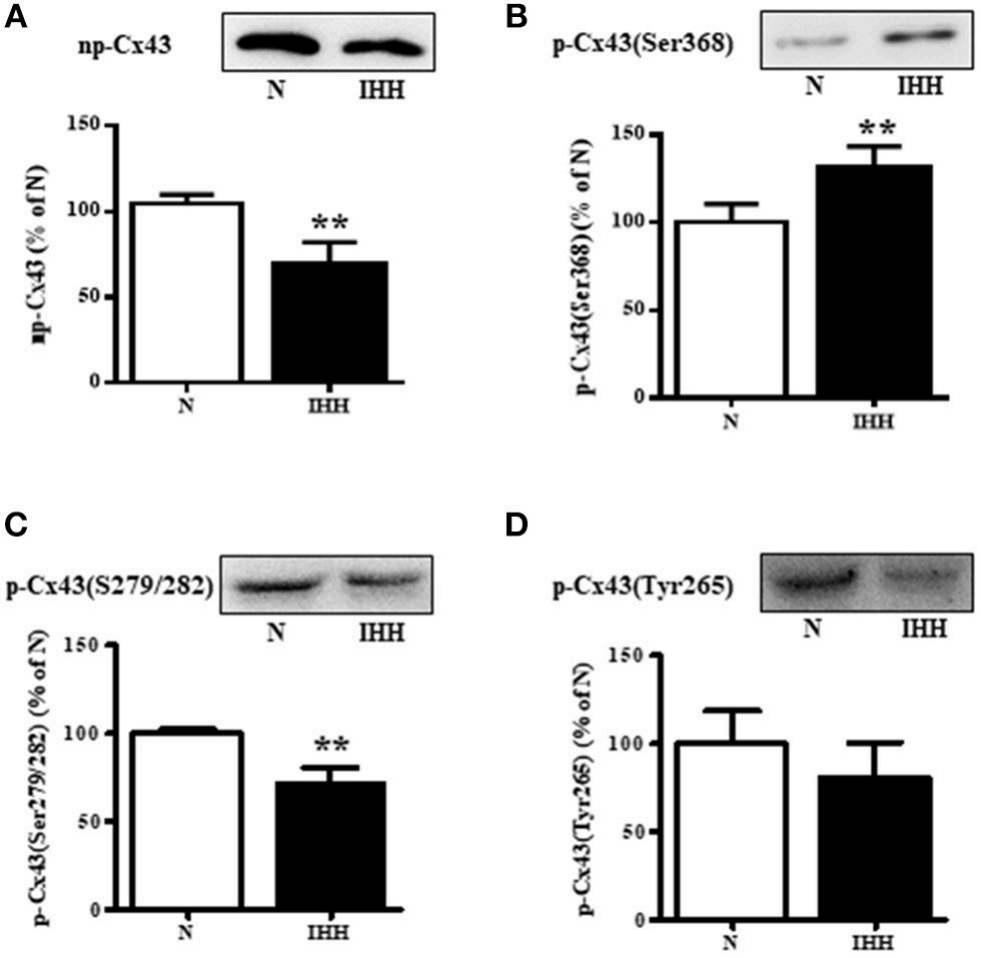
Effect of chronic intermittent hypobaric hypoxia (IHH, black columns) on protein level of non-phosphorylated Cx43 **(A)**, phosphorylated Cx43 at Ser368 **(B)**, phosphorylated Cx43 at Ser279/282 **(C)**, and phosphorylated Cx43 at Tyr265 **(D)** assessed by western blotting in the left ventricular myocardium. N, normoxic group (open columns). Values are mean ± SEM, (*n* = 5 in each group), ^**^P < 0.01.

**Figure 3:**
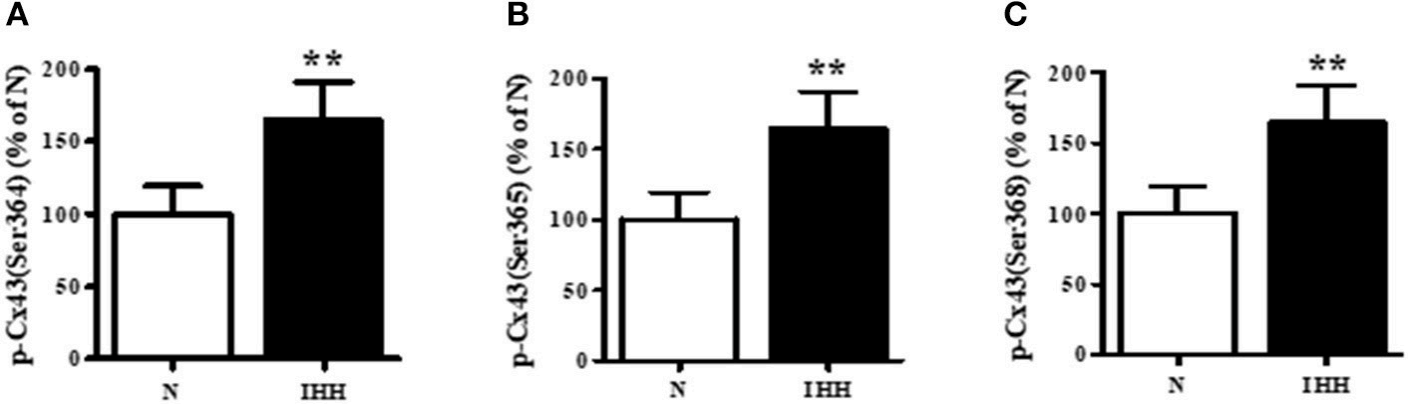
Effect of chronic intermittent hypobaric hypoxia (IHH, black columns) on phosphorylation of Cx43 at Ser364 **(A)**, Ser365 **(B)**, and Ser368 **(C)** assessed by Mass Spectrometry in left ventricular myocardium. N, normoxic group (open columns). Values are mean ± SEM, (*n* = 5 in each group), ^**^P < 0.01.

Total Cx43 expression (t-Cx43) increased by 48% (Figure 1B) and, in parallel, the level of high-phosphorylated P1+P2 forms of t-Cx43 also increased by 56 % (Figures 1A,C) in IHH myocardium compared to normoxic group. Importantly, using specific anti-np-Cx43 antibody we demonstrated a decrease of np-Cx43 expression by 30% in IHH group (Figure 2A). Furthermore, specific antibodies for phosphorylated sites showed that the p-Cx43(Ser368), which increases GJ communication, was elevated in the IHH group by 30% compared to normoxic group (Figure 2B). By contrast, phosphorylation at p-Cx43(Ser279/282), which attenuates intercellular communication, decreased by 27% after IHH (Figure 2C). The phosphorylation at p-Cx43(Tyr265), which may contribute to internalization or distribution of Cx43, did not change (Figure 2D). Additionally, MS analyses revealed that IHH induced increased phosphorylation of p-Cx43(Ser364, Ser365) and confirmed the increase of p-Ser368 (Figures 3A–C, respectively).

### Expression of Cx43 Upstream Kinases

**Figure 4:**
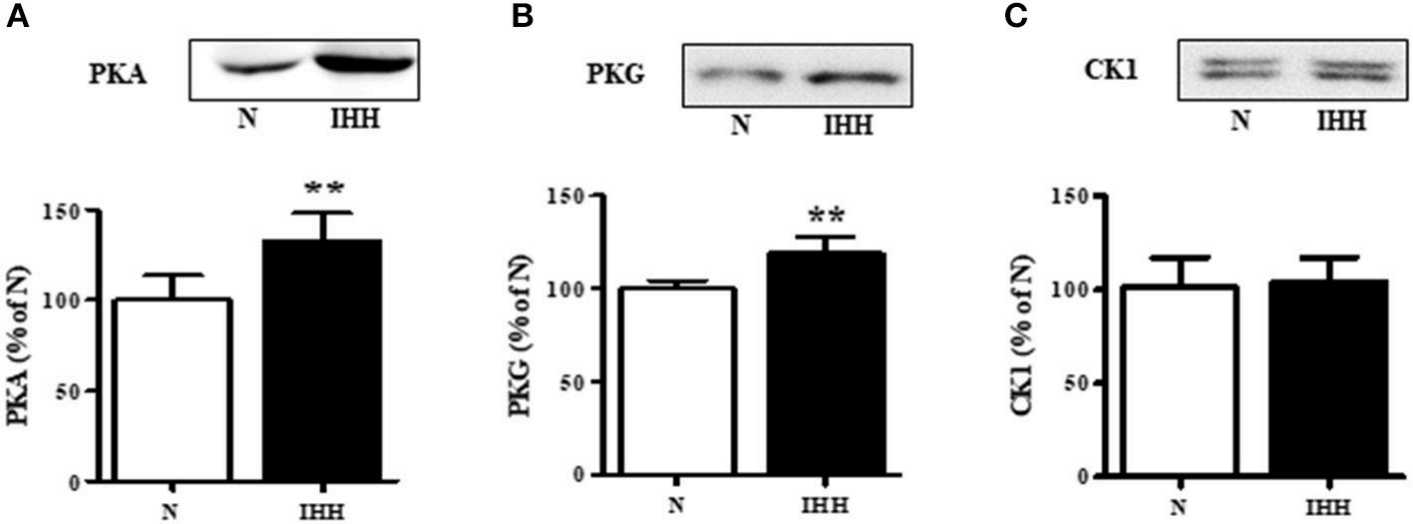
Effect of chronic intermittent hypobaric hypoxia (IHH, black columns) on protein expression of Cx43 upstream protein kinases: protein kinase A (PKA) **(A)**, protein kinase G (PKG) **(B)**, and casein-kinase 1 (CK1) **(C)** assessed by western blotting in left ventricular myocardium. N, normoxic group (open columns). Values are mean ± SEM, (*n* = 5 in each group), ^**^P < 0.01.

With respect to upstream kinases affecting Cx43 function, we showed that in the IHH group, the expression of PKA and PKG increased by 33 and 19%, respectively (Figures 4A,B), whereas there was no change in the expression of CK1 compared to normoxic group (Figure 4C).

### Redistribution of t-Cx43 and p-Cx43(Ser368) to “end to end” Junctions

**Figure 5:**
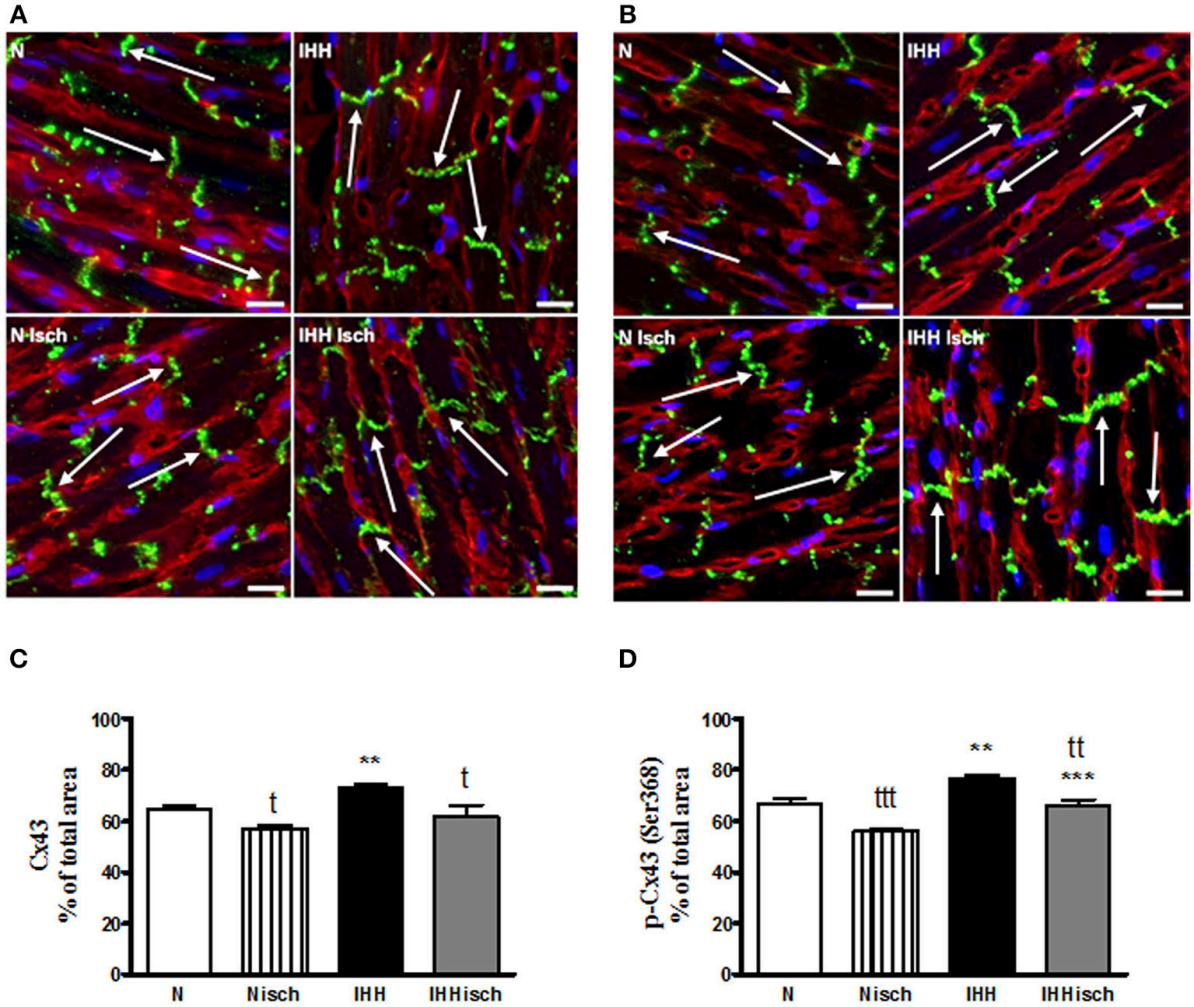
Effect of chronic intermittent hypobaric hypoxia (IHH) on distribution of total Cx43. The representative micrograph of longitudinal cryosections demonstrates distribution of t-Cx43 **(A)** and p-Cx43(Ser368) **(C)** after 10-min regional ischemia in normoxic (N Isch) and hypoxic (IHH Isch) left ventricular myocardium compared to control groups not subjected to ischemia (N, IHH). The green color corresponds to specific Cx43 staining, red color represents sarcolemma (counterstained by WGA) and the blue color indicates nuclei DAPI staining. Note the positive Cx43 staining (arrows) located predominantly at the intercalated discs, i.e., “end to end” junctions of the cardiomyocytes. The proportion of t-Cx43 and p-Cx43(Ser368) at “end to end” junctions in normoxic (N) and hypoxic (IHH) groups under control conditions and after ischemia (Isch) **(B,D)** is expressed as a percentage of total junctions. Values are mean ± SEM, (*n* = 6 in each group), ^*^ vs. corresponding normoxic group; ^t^ vs. corresponding non-ischemic group; ^t^*P* ≤ 0.05, ^**^/^tt^*P* ≤ 0.01, ^***^/^ttt^*P* ≤ 0.001. Scale bar represents 20 μm.

Quantitative immunofluorescence analysis revealed that t-Cx43 was located predominantly at “*end to end”* junctions (Figures 5A,B). IHH increased the area of t-Cx43 at “*end to end”* junctions by 10% compared to normoxic group (Figures 5A,B). Similarly, p-Cx43(Ser368) predominantly localized at “*end to end”* junctions under normoxia (Figures 5C,D), was increased at this location by 10% after IHH (Figures 5C,D). Whereas, 10-min ischemia decreased the proportion of t-Cx43 at “*end to end”* junctions (Figures 5A,B), the significant difference in p-Cx43(Ser368) localization between IHH and N groups persisted (Figures 5C,D). Because the area of “*end to end*” junctions was calculated as a percentage of the total junctions, the “side to side” area changed reciprocally.

### Fatty Acid Composition of Total Phospholipids

**Figure 6:**
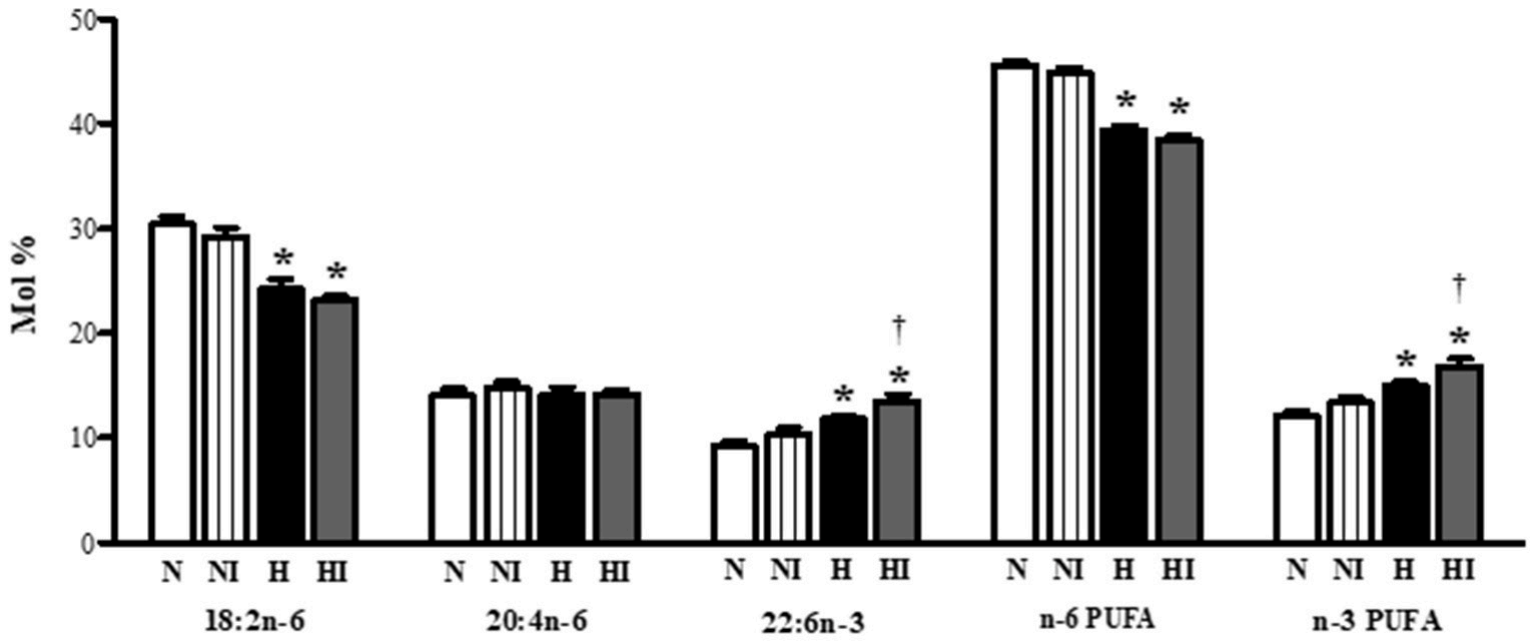
Effect of chronic intermittent hypobaric hypoxia on main polyunsaturated fatty acids [linoleic acid (18:2n−6), arachidonic acid (20:4n−6), docosahexaeonic acid (22:6n−3)], total n−6 PUFA and total n−3 PUFA proportion in total phospholipids in the left ventricular myocardium of normoxic (N) and hypoxic (H) rats under control (non-ischemic) conditions and after 10-min regional ischemia (NI and HI, respectively. Values are mean ± SEM, (*n* = 7 in each group), *P < 0.05 vs. normoxia, †*P* < 0.05 vs. corresponding control group.

Subsequently, we also analyzed the effect of IHH and 10-min ischemia on PUFA proportion in phospholipids (Figure 6). IHH increased the proportion of docosahexaenoic acid (22:6n-3) and total n-3 PUFA, which was accompanied by a decrease of linoleic acid (18:2n-6). The proportion of arachidonic acid (20:4n-6) did not change. While ischemia had no influence on normoxic group, it significantly enhanced n-3 PUFA proportion in IHH group by a further 13% due to the rise of docoshexaenoic acid (Figure 6).

### Ischemic Ventricular Arrhythmias

**Figure 7:**
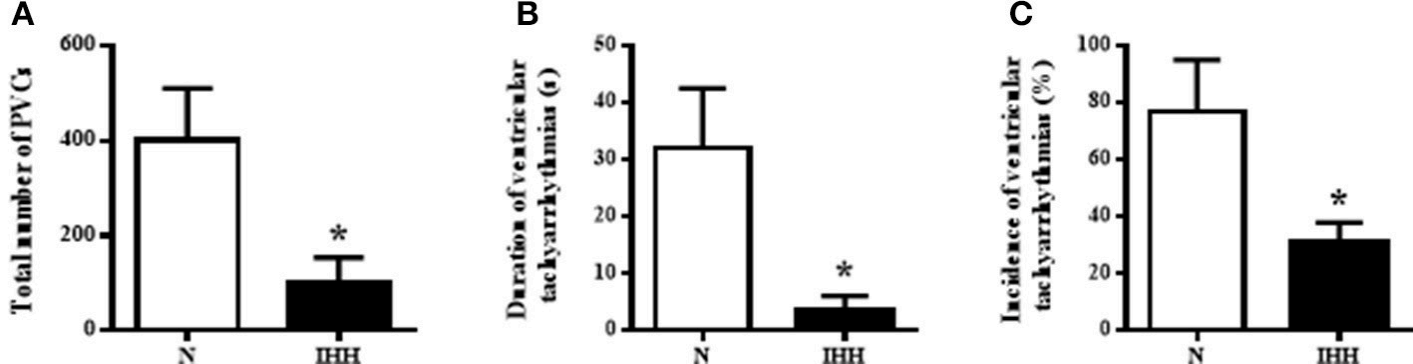
Effect of chronic intermittent hypobaric hypoxia (IHH, black columns) on the incidence of ventricular tachyarrhythmias (tachycardia and fibrillation) **(A)**, total duration of ventricular tachyarrhythmias **(B)** and total number of premature ventricular complexes (PVCs) occurring as singles, salvos and tachycardia **(C)** during 10-min regional ischemia. N, normoxic group (open columns). Values are mean ± SEM, (*n* = 13 in each group), ^*^P < 0.05.

The incidence of ventricular tachyarrhythmias during 10-min coronary occlusion was significantly lower in rats adapted to IHH than in their normoxic counterparts (Figure 7A). IHH also significantly reduced total duration of ventricular tachyarrhythmias (Figure 7B) as well as the total number of premature ventricular complexes occurring as singles, salvos or tachycardia (Figure 7C).

## Discussion

In the present study we demonstrated the association of anti-arrhythmic phenotype of IHH rats with increased myocardial t-Cx43 protein level and its phosphorylation, as well as enhanced location of both t-Cx43 and p-Cx43(Ser368) at “*end to end”* junctions; the latter effect persisted even after 10-min ischemia. Importantly, brief ischemia further increased the already high proportion of n-3 PUFA in membrane phospholipids induced by IHH. This observation is in agreement with enhanced endogenous protection against life-threatening I/R arrhythmias described earlier in the same experimental model of IHH (15, 16, 31).

The elevated protein expression of t-Cx43 and its phosphorylated forms due to IHH is consistent with studies suggesting that protein up-regulation of myocardial Cx43 is linked to protection from arrhythmias [for review see (7)]. It is noteworthy that our experimental model of chronic IHH differs from models of intermittent hypoxia consisting of multiple cycles of brief severe hypoxia and reoxygenation episodes used to simulate obstructive sleep apnoea (OSA) syndrome as an important risk factor of cardiovascular diseases. It has been shown that OSA results in reduction and remodeling of myocardial Cx40 and Cx43, which may contribute to arrhythmogenic substrate (32). Other maladaptive responses resulting in increased propensity to arrhythmias, such as hypertension (33), altered thyroid status (34), and doxorubicin-induced cardiomyopathy (35) are also accompanied by down-regulation of Cx43. Similarly, conditional Cx43 knockout facilitates pro-arrhythmia state and contributes to cardiac contractile dysfunction (1, 36, 37).

We have further demonstrated that IHH affected the phosphorylation status of Cx43 at four sites: Ser364, Ser365, Ser368, and Ser279/282 belonging to upstream PKA, PKC, and MAPK, respectively. Additionally, protein kinase B/Akt, CK1, and v-Src tyrosine kinase are involved in controlling Cx43 phosphorylation [reviewed by (38, 39)]. Post-translational modifications of Cx43 play an important role in the regulation of GJ assembly, conductivity and permeability, depending on the phosphorylation site and the executing upstream kinases (40).

PKA upregulation by IHH was accompanied by PKA-targeted phosphorylation of Cx43 at Ser364, Ser365 which accelerates Cx43 assembly and conductivity (41, 42). Moreover, phosphorylation at Cx43(Ser365) is considered as a gate-keeper of these events (42). On the other hand, IHH upregulated PKG which has been shown to attenuate conductivity (43). Contrary to that, neither the expression of CK1 nor phosphorylation of targeting phospho-sites at Ser325/Ser328/Ser330 was affected by IHH suggesting that IHH did not influence the main pathway responsible for the redistribution of Cx43 from the plasma membrane into GJs (44, 45). Thus enhancement of both PKA and PKG might be involved in increased Cx43 trafficking, assembly and moderation of conductivity induced by IHH.

Both MS analyses and western blotting of IHH myocardium revealed increased phosphorylation of Cx43(Ser368) which is mostly attributed to PKCε (46). In line with this view, Hlaváčková et al. (47) reported an increased p-PKCε(Ser729)/PKCε ratio indicating enhanced activation of the enzyme under IHH conditions. Importantly, the increased level of p-Cx43(Ser368) by PKCε, stabilizes GJs in intercalated discs, prevents lateralization and attenuates Cx43 channel conductivity (48). Accordingly it prevents inducible ventricular fibrillation (49). PKCε-related phosphorylation of Cx43 at Ser368 may be additionally fine-tuned by suppression of Cx43 phosphorylation at Tyr265 and Ser279/282 sites; the latter was observed in the present study. Thus, PKCε activation can be implicated in IHH-related suppression of arrhythmia susceptibility, likely due to rectifying conductance in “*end to end*“ junctions and reducing conduction in favor of electrical stability.

As mentioned before, we found that the level of MAPK-mediated p-Cx43(Ser279/282), possessing inhibiting effect on GJs, decreased in IHH group. This suggests that IHH attenuates MAPK-controlled internalization and subsequent degradation of Cx43 by endocytosis and thus stabilization of Cx43 channel gating and conductivity of GJs (40, 50). Besides that, phosphorylation of Cx43(Tyr265), mediated by v-Src, did not change in IHH myocardium compared to normoxic controls. It was shown that the v-Src phosphorylation of Cx43 at Tyr247/Tyr265 inhibits GJ communication and may cause their disassembly (51).

It has been reported that Akt activity controls GJ stability and participates in formation of larger and stable GJs (52). Direct inhibition of Akt leads to the loss of GJ by internalization. Phosphorylation of Cx43 at S373 by Akt appears to control GJ size through inhibition of the interaction of Cx43 with ZO-1 (53). Interestingly, it has been demonstrated that under conditions of injury (wound healing) or growth factor treatment, Cx43 becomes sequentially phosphorylated by Akt (5–15 min post treatment on p-Ser373), MAPK (at 15–30 min on p-Ser279/S282) and v-Src (at 30 min extending for hours on p-Tyr247) resulting in sequential changes in GJs, including increased size followed by inhibition of GJ communication and their internalization from the plasma membrane (38). Importantly, we have documented highly activated Akt after 10-min ischemia in chronically hypoxic heart (54).

In line with a number of previous studies, we observed decreased incidence and duration of ventricular tachyarrhythmias as well as reduced total number of premature ventricular complexes during 10-min ischemia in rats adapted to IHH. This IHH-induced anti-arrhythmic protection was associated with, changes in Cx43 distribution. Quantitative image analysis revealed slightly but significantly increased proportion of both t-Cx43 and p-Cx43(Ser368) at “*end to end”* GJs in IHH myocardium. Whereas, 10-min ischemia basically decreased the proportion of Cx43 at “*end to end”* junctions, the difference in p-Cx43(Ser368) localization between IHH and N groups persisted. This can be considered as a benefit of IHH adaptation that may participate in the decreased susceptibility of hearts to ischemia-induced arrhythmias. In line with this, anti-arrhythmic ischemic preconditioning prolonged the time to plateau in electrical uncoupling, translocation of Cx43 from GJs to the cytosol and dephosphorylation of Cx43 in rat hearts (13). On the other hand, abnormal Cx43 lateralization, due to prolonged ischemia, was accompanied by Cx43 dephosphorylation (55). Furthermore, reduced overall expression of Cx43 as well as increased lateralization accelerated the onset, incidence, frequency, and duration of ventricular arrhythmias after coronary artery occlusion (56). High lateral “*side to side”* GJ conductance increased the susceptibility to conduction block providing a substrate for arrhythmias (6, 57–59). Based on the above, we can assume that increased phosphorylation of PKA and PKC target phospho-sites of Cx43 accompanied by the decrease in MAPK targeting Cx43 inhibiting sites, as well the decrease of lateralization, contributes to IHH elicited anti-arrhythmic phenotype possibly based on moderating permeability and rectifying “*end to end*” conductivity.

In general, cardioprotective action of n-3 PUFA has been widely documented [reviewed by (60, 61)]. Their anti-arrhythmic effect may depend on the integrity and lipid composition of membranes, affecting conductivity e.g., by reduction of membrane electrical excitability and activity of voltage-dependent sodium channels in cardiomyocytes (62, 63) as well as by modulation of calcium channels (64) and possibly through shortening of the action potential (65). It has been also reported that n-3 PUFA improved electrical remodeling, increased Cx43 expression and reduced arrhythmias in hypertensive rat hearts (66, 67) suggesting tight relation between anti-arrhythmic effect of Cx43 and n-3 PUFA. We have shown previously that IHH increased the proportion of n-3 PUFA in heart phospholipids (19). The n-3 PUFA dietary supplementation of rats during adaptation to IHH resulted in a cumulative anti-arrhythmic effect which almost completely eliminated ventricular arrhythmias induced by I/R insult (68). The present study showed that brief ischemia even further enhanced the already high proportion of n-3 PUFA in membrane phospholipids of IHH hearts, which is in line with increased p-Cx43(Ser368) level at “*end to end”* GJs.

In conclusion, we have demonstrated that chronic IHH, which induces strong protection against arrhythmias occurring during acute I/R insult, is associated with increased expression, phosphorylation and redistribution of myocardial Cx43 as well as with increased anti-arrhythmic n-3 PUFA proportion in heart phospholipids persisting after brief ischemia.

## Ethics Statement

The maintenance and handling of experimental animals was in accordance with the Guide for the Care and Use of Laboratory Animals published by the US National Institutes of Health (NIH Publication No. 85–23, revised 1996). The experimental protocol was approved by the Animal Care and Use Committee of the Institute of Physiology of the Czech Academy of Sciences.

## Author Contributions

JK performed major part of experiments, interpreted data, drafted manuscript. BE image quantitative analyses. KH performed experiments. JN hypoxic model, performed experiments. OS image quantitative analyses. JJ fatty acid analyses. MV fatty acid analyses. PV, BS, TE and MH performed experiments. DH performed experiments, prepared images. NT performed experiments, edited manuscript. FK hypoxic model, edited manuscript. ON fatty acid analyses—interpreted data, edited manuscript, and finalized manuscript. JZ experimental design, performed experiments, interpreted data, finalized manuscript.

### Conflict of Interest Statement

The authors declare that the research was conducted in the absence of any commercial or financial relationships that could be construed as a potential conflict of interest. The handling Editor declared a shared affiliation at the time of review, though no other collaboration, with several of the authors, JK, BE, OS, JJ, MV, PV, DH, ON and JZ.
